# Thermoplastic Network Formation as a Method for Stabilizing Salt Hydrate Particles

**DOI:** 10.3390/molecules30234519

**Published:** 2025-11-22

**Authors:** Elena Averina, Hartmut Fischer, Olaf C. G. Adan, Hendrik P. Huinink

**Affiliations:** 1 Department of Applied Physics and Science Education, Eindhoven University of Technology, Den Dolech 2, 5600 MB Eindhoven, The Netherlands; 2 Eindhoven Institute for Renewable Energy Systems, Eindhoven University of Technology, P.O. Box 513, 5600 MB Eindhoven, The Netherlands; 3 TNO Materials Solutions, High Tech Campus 25, 5656 AE Eindhoven, The Netherlands

**Keywords:** thermochemical energy storage, salt hydrates, composites, stabilization, thermoplastic polymers

## Abstract

Thermochemical energy storage (TCES) utilizes chemical reactions to store thermal energy, offering a promising solution for efficient energy management. However, a significant challenge in application of TCES materials, particularly with crystal-to-crystal chemical transformations, is the mechanical degradation of reactive particles during repeated cycles connected with the constant re-modeling of crystals due to consecutive hydration–dehydration steps. This degradation leads to increased pressure drops in packed beds due to swelling and fracturing of salt particles, complicating their practical application. To address this issue, this study investigates the effect of a polymeric network as stabilizing element within TCES particles to enhance mechanical stability. Using potassium carbonate hydrate (K_2_CO_3_·1.5H_2_O) as a model thermochemical material and thermoplastic polymers for reinforcement, composite particles were developed to resist disintegration over multiple cycles. The incorporation of polymeric networks from polyamide (PA11), polyetherimide (PEI) and polyvinylidene fluoride (PVDF) resulted in improved mechanical properties at relatively high porosity, which contributes to higher hydration rate. The developed stabilization method is compatible with existing scalable particle production methods like tableting and compacting.

## 1. Introduction

Salt hydrates are widely recognized for their potential in thermal energy storage systems due to their high energy density and capability to undergo reversible hydration and dehydration cycles [1,2,3]. An additional advantage of salt hydrate systems is their suitability for heat storage [4]. These materials can efficiently store and release heat, making them ideal for applications in renewable energy systems and thermal management technologies.

However, the practical application of salt hydrates is often limited by mechanical degradation during repeated hydration cycles [5]. This degradation occurs as the salt particles undergo volumetric changes, leading to the formation of cracks and a loss of structural integrity, ultimately reducing the performance and lifespan of the storage material [6,7].

Potassium carbonate sesquihydrate was selected as a model process as it was shown to be promising for thermochemical energy storage [3,8] due to its low cost, low toxicity, and favorable thermodynamic parameters of the reaction

(1) $${K_{2}CO_{3}\cdot {1.5H}_{2}O}_{\left(s\right)}⇆{K_{2}CO_{3}}_{\left(s\right)}+{{1.5H}_{2}O}_{\left(gas\right)}$$

Despite the advantages, it undergoes considerable shrinkage and swelling during cycles [9,10], and can be readily put into deliquescence.

To mitigate these issues, researchers have explored various methods to stabilize salt hydrate particles such as composite formation and encapsulation [11]. One approach involves using a porous matrix by impregnating the salt into materials as vermiculite [10], zeolites [12], and polymeric foams [13,14,15]. Other investigated stabilizing methods are using polymeric binders, such as poly (dimethyl siloxane) (PDMS) [16] or polymeric fibers as stabilizing additives [17]. Encapsulation approaches have demonstrated long-cycle stability but often involve complex, non-scalable processing [18,19]. More recent work has targeted K_2_CO_3_ granule stability and membrane/encapsulation concepts to improve robustness under cycling [20].

In contrast, the present study introduces a solvent-free, melt-processing route to form thermoplastic polymer networks directly within salt tablets.

While progress has been achieved in enhancing the mechanical durability of TCES materials, most existing stabilization strategies still present key challenges. Porous matrices often involve a trade-off between energy storage density and temperature lift [15]; polymer binders or coatings may limit scalability due to solvent-based or reactive processing; and encapsulation routes typically require complex fabrication steps. To enable industrial implementation, stabilization methods must be compatible with simple, low-cost, and scalable manufacturing processes such as tableting and compaction.

Using polymeric networks as a stabilization method may provide mechanical reinforcement while maintaining a high concentration of active salt, potentially minimizing the reduction in energy storage density. Unlike polymeric binders, which typically coat or adhere and may interfere with the active material, polymeric networks serve as a structural framework. These networks act more like a skeletal scaffold—they form a continuous, interconnected structure throughout the tablet. This allows the salt to remain chemically and functionally accessible while the polymer network provides mechanical stability. By forming a flexible and interconnected network within the salt tablet, polymers can help distribute mechanical stresses more effectively, reducing mechanical degradation without excessively restricting the volumetric changes necessary for efficient thermal cycling.

In this work, we investigate the potential of using thermoplastic polymer particles to form polymeric networks within salt tablets as a stabilization approach, which is compatible with widely used methods to produce particles from powder such as tableting and compaction. Such compatibility will allow us to upscale the TCM production quickly to industrial levels. Thermoplastic materials can be processed through straightforward techniques such as compression molding or extrusion, offering a scalable and reproducible method for stabilizing salt hydrates. Instead of focusing solely on specific polymers, this study aims to evaluate which polymer properties are most favorable for stabilizing salt hydrates, considering factors such as mechanical support, chemical compatibility, thermal stability and processibility. By examining how different polymers interact with salt hydrates, we seek to provide insights into the suitability of polymer particles and their transformation to internally stabilizing networks for thermal energy storage applications.

Among the various polymers available, polyamide 11 (PA11), polyvinylidene fluoride (PVDF), and polyetherimide (PEI) have been selected due to their properties such as thermal stability in the temperature range of TCES particles hydration/dehydration cycling, elongation at break, allowing mechanical stabilization of TCM particles during swelling. While the selected polymers fall within the targeted property range, they also offer variability, enabling the investigation of a broader spectrum of material behaviors.

PA11 is known for its high tensile strength and flexibility, which make it suitable for forming robust networks at relatively low melting temperatures. Additionally, PA11′s bio-based origin and low environmental impact make it an attractive choice for sustainable applications [21]. PVDF, on the other hand, is known for its chemical resistance, thermal stability, and mechanical strength, which can contribute to maintaining the integrity of the salt hydrate tablets under harsh conditions, such as exposure to alkaline environments [22]. PEI provides rigidity, enhancing the mechanical stability of composite materials [23,24] and offering insights into more rigid network suitability for this application.

In this investigation, we explore the use of PA11, PVDF, and PEI to stabilize salt hydrate particles through the formation of polymeric networks within the tablets with the aim to evaluate the mechanical stability, structural integrity, and hydration–dehydration performance of these composite materials. By examining the interactions between different polymeric properties and salt hydrate behavior, we seek to provide insights into the suitability of polymer-based stabilization methods for thermal energy storage applications.

## 2. Materials and Methods

### 2.1. Materials

K_2_CO_3_·1.5H_2_O was supplied by Evonik Functional Solutions GmbH in two particle size distributions: 50 μm and 250 μm (average). Initially, the salt was examined without further processing. For subsequent investigations, the 250 μm variant was sieved to remove particles larger than 300 μm, preventing the formation of excessively large crystals that could disrupt the polymer matrix. The fraction with particle sizes ≤ 300 μm was used for further studies. Additionally, a 300–500 μm fraction was separately analyzed, as discussed in the relevant section.

Polyamide (PA11) powder for 3D printing, with an average particle size of 58 μm and bulk density 1.1 g/cm^3^, was obtained from Sinterit (Krakow, Poland). Polyetherimide (PEI) (average particle size 25 μm, bulk density 1.17 g/cm^3^) and polyvinylidene fluoride (PVDF) (average particle size 25 μm, bulk density 1.05 g/cm^3^) powders were sourced from Chemazone (Leduc, AB, Canada). The particle sizes and bulk densities provided by the suppliers.

### 2.2. Preparation

Potassium sesquihydrate powder and polymer powder were mixed by using laboratory roller mixer (RM-200, Janke & Kunkel, Staufen im Breisgau, Germany) in various ratios until homogeneous mixture, followed by placing the mixture to the mold with diameter 12.5 mm then pressed using a PO-Weber PW-40 2 column press (Remshalden, Germany) with manufacturing pressure 1 kbar. The pressure was manually applied within approximately 10 s and held constant for 30 s for each tablet to ensure uniform compaction.

The obtained tablets were placed in the oven under following conditions: for K_2_CO_3_/PA11 at 210 °C for 1 h, for K_2_CO_3_/PEI at 315 °C for 15 min, for K_2_CO_3_/PVDF at 250 °C for 30 min. The temperatures above the melting point were selected empirically, as described later in Section 3.2. At these temperatures, the polymers melted into a sufficiently mobile liquid capable of forming a network within the salt tablet. All heating procedures were carried out under ambient atmosphere. Tablet weight and dimensions were measured immediately after the heating stage was completed using a digital caliper (Mitutoyo, Kawasaki, Japan).

**Figure 1:**
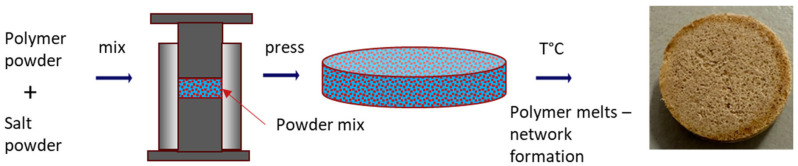
Schematic preparation method.

To visually observe the formation of polymeric network, salt was dissolved to reveal the skeleton of the network (Figure 1).

### 2.3. Characterization

#### 2.3.1. Compression Test

A Shimazu AGS-X autograph tensile tester equipped with Spherically Seated Compression Plates (Shimadzu Corporation, Kyoto, Japan) was used for testing the compression strength. The cylindric tablets were put perpendicularly to the compression plane. The testing speed was 0.025 mm/min and the force between the plates was registered by a force sensor with the limit of 200 N. As soon as the compression force sharply reduced by more than 10% the compression limit was deemed to be achieved.

#### 2.3.2. Scanning Electron Microscopy (SEM)

SEM analysis was performed on a FEI Quanta 600 scanning electron microscope (Thermo Fisher Scientific, Waltham, MA, USA) using 2 kV and high vacuum as well as 10 kV and low vacuum measurements to reduce charge accumulation on the sample. Prior to the imaging the samples containing the anhydrous salt were cryogenically fractured by freezing the samples in liquid nitrogen. This procedure gave samples with a clear fracture plane, facilitating imaging of the internal structure of the composites.

#### 2.3.3. Cycling Ang Kinetic Measurement

The hydration/dehydration cycling was performed directly after the formulation of tablet starting with first dehydration until the mass of tablet reaches the calculated mass of fully anhydrous state as described below, followed by hydration until stable mass of hydrous tablet. The kinetics of hydration was measured ex situ by putting the dry single tablets of both K_2_CO_3_ and K_2_CO_3_/polymer into a desiccator over a saturated MgCl_2_ solution, which produced relative humidity 33% at 20 ± 1 °C [25]. The relative humidity inside the desiccator (33%) was monitored using an RH2 Relative Humidity Meter with External Probe (Checkline Europe, Bad Bentheim, Germany). To ensure a homogenous distribution of water vapor a small ventilator was placed inside the desiccator. This setup enabled continuous verification of humidity stability during the cycling and kinetic measurements. The mass m(t) [g] at a certain moment was recorded using a precision analytical balance (Mettler Toledo MS205DU, Greifensee, Switzerland) by briefly taking a tablet out from the desiccator and putting it back immediately after weighing. The kinetic curves were derived from periodic weighing of each tablet, then calculating conversion α of reaction (2) as follows:

(2) $$\alpha (t)=\frac{m\left(t\right)-m(d)}{m\left(h\right)-m(d)}$$

where m(t) [g] is mass of a tablet at the moment of measurement, m(d) [g] is the mass of a fully anhydrous tablet and m(h) [g] is the mass of a fully hydrous tablet. The theoretical mass of anhydrous tablet was calculated by multiplying the mass of hydrated tablet by mass fraction of anhydrous K_2_CO_3_ calculated from molar masses:

(3) $$m\left(d\right)=m(h)\frac{M_{K_{2}CO_{3}}}{M_{K_{2}CO_{3}\cdot 1.5H_{2}O}}$$

For measuring dehydration kinetics, fully hydrated tablets were put in an oven at 130 °C instead of the desiccator. In total, four tablets of K_2_CO_3_ and K_2_CO_3_/polymer were characterized simultaneously, the kinetic curves were averaged.

The cycling conditions (relative humidity 33% at 20 °C) were chosen to ensure reproducible and comparable measurements across samples. Similar parameters have been employed in previous TCES investigations [9,16], as they enable clear evaluation of intrinsic hydration and dehydration behavior while minimizing external effects such as vapor flow and temperature variations. This controlled approach allows for consistent comparison between polymer systems.

#### 2.3.4. Porosity Measurement

The porosity of tablets was determined from mass m [g], weight content of the salt ω [-] and geometric parameters (diameter d [mm] and thickness t [mm]) measured by means of a digital caliper (Mitutoyo, Kawasaki-shi, Japan).

The porosity in dehydrated ε_d_ [-] and hydrated ε_h_ [-] states was calculated based on previous work [17] as follows:

Considering bulk densities of the salt (ρ_h_ = 2.18 g/cm^3^ for K_2_CO_3_·1.5H_2_O and ρ_a_ = 2.43 g/cm^3^ for K_2_CO_3_ [26,27]) and bulk density of polymer. The calculation was carried out as follows:

(4) $$\varepsilon_{h}=1-\frac{V_{skeletal}}{V}=1-4\frac{m}{\pi \rho_{h}d^{2}t}\left[\omega +(1-\omega )\frac{\rho_{h}}{\rho_{p}}\right]$$

(5) $$\varepsilon_{d}=1-\frac{V_{skeletal}}{V}=1-4\frac{m}{\pi \rho_{a}d^{2}t}\left[\omega +(1-\omega )\frac{\rho_{a}}{\rho_{p}}\right]$$

In these equations, bulk densities of the salt are ρ_h_ = 2.18 g/cm^3^ (K_2_CO_3_·1.5H_2_O) and ρ_a_ = 2.43 g/cm^3^ (anhydrous K_2_CO_3_) [21,22]). The bulk densities of the polymeric phase ρ_p_ is 1.1 g/cm^3^ (PA11), 1.17 g/cm^3^ (PEI) or 1.05 g/cm^3^ (PVDF).

## 3. Results and Discussion

### 3.1. Network Formation Using PA-11

A concept proof was performed using polyamide-11 particles designed for 3D printing application. PA11 has a relatively low melting temperature (200 °C) due to its high methylene/amide group ratio [21,28], at the same time exhibiting strong mechanical performance, print quality, and physicochemical properties of PA11 powder (~58 μm) used in 3D printing, highlighting PA11′s tensile strength and low coefficient of friction, making it a good candidate for industrial applications [27].

To determine the optimal salt particle size for network formation, a preliminary test was conducted using two configurations of K_2_CO_3_·1.5H_2_O provided by Evonik Functional Solutions GmbH: a fine powder with an average particle size of 50 μm and a coarser powder with an average particle size of 250 μm.

Two types of tablets were prepared as described in Section 2.2. The first type contained 10 wt% PA11 powder and 90 wt% fine salt powder (50 μm), while the second type contained 10 wt% PA11 powder and 90 wt% coarse salt powder (250 μm). Both types of tablets were subjected to hydration/dehydration cycling, as described in Section 2.3.3.

**Figure 2:**
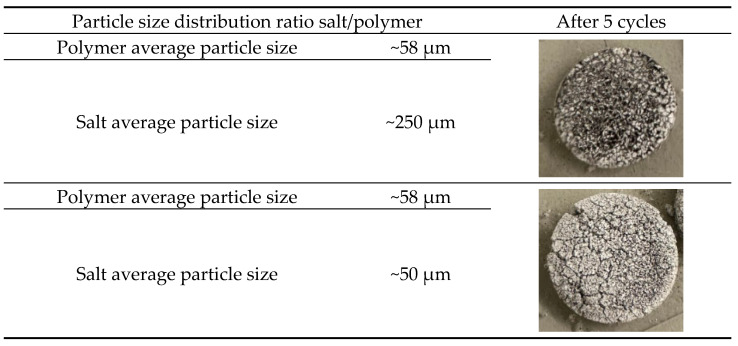
K_2_CO_3_/PA11_10 wt% tablets: comparison of network stability between tablets prepared with K_2_CO_3_ of average particle sizes 250 μm and 58 μm after 5 cycles (average particle sizes provided by the supplier).

The visual comparison of tablets allows to conclude that network formation was significantly weaker when the salt particles were similar in size to the polymer particles, resulting in partial tablet disintegration and cracking after 5 cycles. In contrast, samples prepared with salt particles larger than the polymer particles exhibited more stable network formation without visible cracks and tablet remaining intact (Figure 2).

This outcome can be attributed to the difference in particle sizes between the salt and the polymer. When the polymer particles are much smaller than the salt particles, the polymer particles fill up the void space between the salt particles more efficiently. Therefore, a more continuous network of polymeric particles is present. In contrast, smaller salt particles have a higher surface area, requiring more polymer for adequate coverage. If the polymer content is insufficient, this can result in weaker network structures [28]. Based on these findings, the fine salt powder (50 μm) was excluded from further investigation. Instead, the 250 μm salt, which demonstrated more stable network formation with PA11 powder (58 μm), was selected. To ensure uniform polymer network formation, this salt was sieved to remove particles larger than 300 μm, preventing the formation of excessively large crystals that could disrupt the polymer matrix. Further investigation was conducted using ≤300 μm salt fraction.

**Figure 3:**
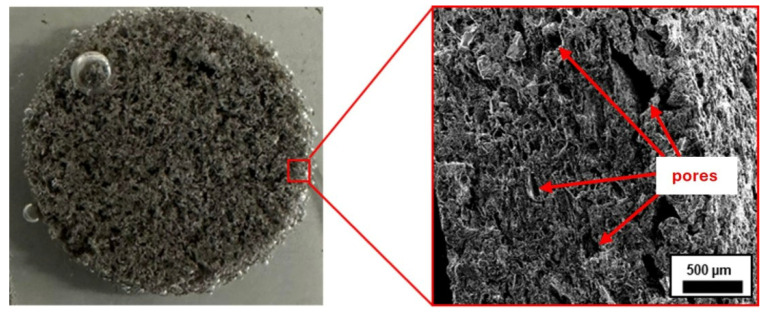
Picture of PA11 network after dissolving K_2_CO_3_ (**left**), SEM image of PA11 network after dissolving K_2_CO_3_ (**right**).

To demonstrate that melting polymeric powder inside the tablet provides an intact polymeric network, tablet was repeatedly placed in water until all salt was dissolved and a pure polymeric network was obtained (Figure 3).

As can be seen, the remaining structure appears as a cohesive matrix, indicating the formation of a continuous polymeric phase resistant after salt dissolution. Although the pore sizes vary, they do not disrupt the integrity of the network.

**Figure 4:**
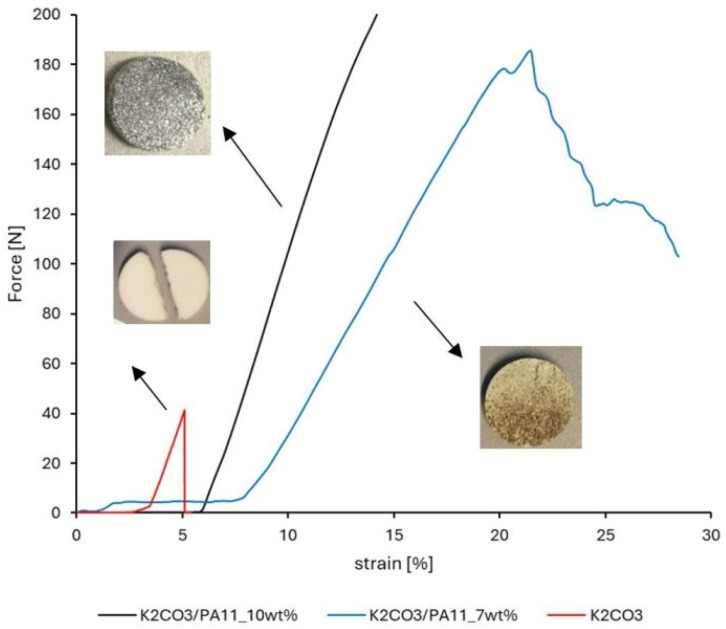
Compression test of pure K_2_CO_3_ (red), K_2_CO_3_ with 7 w% PA11 (blue), K_2_CO_3_ with 10 w% PA11 (black).

The mechanical stability of salt/polymer tablets was evaluated by a compression test. Experiments were performed with tablets having a weight fraction of polymer of 0, 7 and 10% (four to five tablets per each sample). It was observed that increased mechanical stability correlates with increasing the amount of polymer in tablet (Figure 4).

It was determined that the minimal amount of polymeric powder in salt/powder mixture that provides network formation varies from 7 to 10 wt%. A polymer content of 10 wt% was selected for further investigation, as it provided sufficient cohesion to prevent tablet cracking. Further increasing the polymer fraction would reduce the active salt content, while lower polymer fractions did not provide effective stabilization.

### 3.2. Comparing Polymers

For further investigation, different thermoplastic polymers were compared. Thermoplastic polymers were selected based on literature [29,30,31,32,33] (Table 1). The main criteria were melting temperature and elongation at break. As the upper limit of temperature during the cycling varies up to 160 °C [3], it is preferable that melting point of the polymer is above this range. The elongation at break is a parameter, which decides whether a flexible or rigid polymer network can be formed. During tablet volume expansion the flexibility or rigidity can have both positive and negative effects. A rigid network may potentially reduce volume expansion but can lead to crack formation, where a flexible network may potentially keep the tablet intact during swelling.

The selection of polyamide 11 (PA-11), polyetherimide (PEI), and polyvinylidene fluoride (PVDF) as polymer matrices for salt composites via melting-based processing is justified based on their melting temperatures, elongation at break, and water uptake properties, as outlined in Table 1.

While these three polymers were chosen for their properties, the insights gained from their performance can guide the selection of other thermoplastics with similar characteristics for further optimization. This approach enables the development of a mechanically stable, thermally resistant, and adaptable polymer matrix suitable for salt composite applications in thermal energy storage.

A key requirement for the polymer matrix is to remain thermally stable throughout cycling, which involves temperatures up to 160 °C. Therefore, the melting temperature of the selected polymers must be above this range to prevent premature softening or deformation.

PA-1, PEI and PVDF all have melting points sufficiently above 160 °C, ensuring they retain their structural integrity during thermal cycling. PEI, with the highest softening temperature, offers the most thermal stability, whereas PVDF, with a slightly lower melting point, still remains within an acceptable range based on empirical processing observations. Elongation at break is a critical factor for managing tablet volume expansion during hydration and dehydration cycles. PA-11 offers high flexibility, which can help accommodate tablet swelling and reduce crack formation. This makes it a good candidate for systems where mechanical adaptability is beneficial. PEI provides a rigid but not excessively brittle network, which may help in reducing volume expansion while still maintaining some degree of elasticity to withstand stress without immediate failure. PVDF exhibits variable flexibility, depending on processing conditions. Water uptake can influence mechanical stability and long-term durability of the polymer-salt composite. PA-11 allows some moisture absorption, which may help in enhancing adhesion to the salt phase but must be controlled to avoid polymer degradation. PEI provides minimal water uptake, making it more stable in humid environments. PVDF offers a range of water resistance, where lower uptake values make it highly hydrophobic, providing chemical stability and moisture resistance in the composite.

The polymers were chosen to represent distinct classes of thermoplastics, with differing chemical structures and mechanical behavior—from flexible, partially hydrophilic PA-11, to chemically resistant and elastic PVDF, and rigid, highly stable PEI. This diversity allowed a comparative evaluation of polymer influence on network formation, rather than focusing on identifying a single optimal polymer. This study therefore serves as a proof of concept for polymer-assisted stabilization of salt hydrates and provides insights into structure–property relationships for future material optimization in scalable TCES systems. In addition to these properties, the selection of PA-11, PEI, and PVDF was influenced by their availability in powder form within the particle size range of 25–60 µm, comparable to the salt particles (50–250 µm). This was essential for achieving homogeneous mixing and consistent network formation throughout the composite.

**Figure 5:**
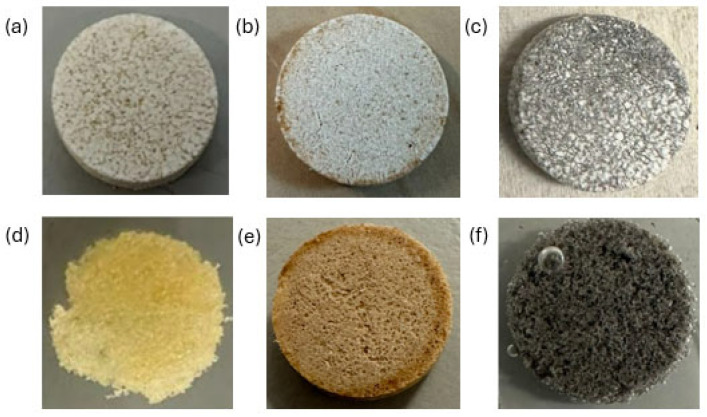
The original K_2_CO_3_/polymer 10 wt% composites: (**a**) PEI; (**b**) PVDF; (**c**) PA11; polymeric networks after dissolving K_2_CO_3_: (**d**) PEI; (**e**) PVDF; (**f**) PA11.

The selected polymers provide variety of properties within selected range in terms of thermal, mechanical, and water absorption properties:

As described in Section 3.1, the polymer content in the tablets was selected as 10 wt%. To ensure a fair comparison between different polymers, all tablet samples were prepared with the same polymer content. The following formulations were prepared as detailed in Section 2.2: K_2_CO_3_/PA11 (10 wt%), K_2_CO_3_/PEI (10 wt%), and K_2_CO_3_/PVDF (10 wt%). To demonstrate intact polymeric networks, tablets were repeatedly placed in water until all salt was dissolved and a pure polymeric networks were obtained. The polymeric networks are shown in Figure 5.

Further investigation of the tablets was performed comparing the following tablet samples: K_2_CO_3_/PA11 10 wt%, K_2_CO_3_/PEI 10 wt%, K_2_CO_3_/PVDF 10 wt%, prepared as described in Section 2.2. The investigation of tablets properties such as mechanical stability, porosity, kinetics was conducted measuring characteristics over hydration/dehydration cycling, performed as described in Section 2.3.3.

**Figure 6:**
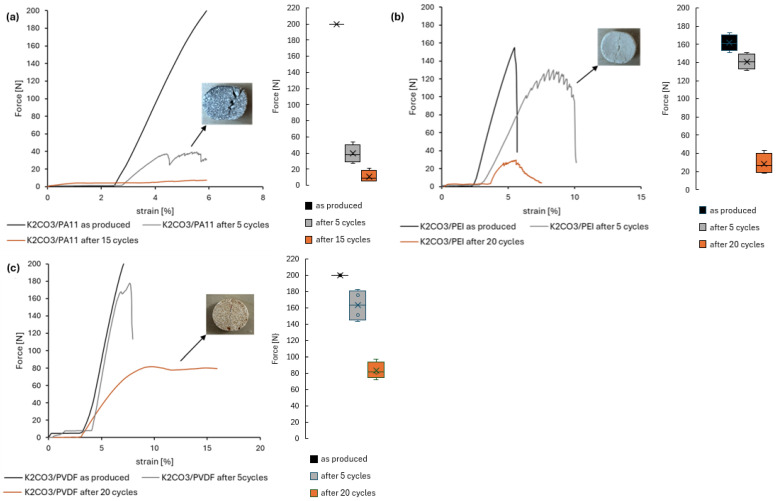
Compression test K_2_CO_3_/PA11 (**a**), K_2_CO_3_/PEI (**b**), K_2_CO_3_/PVDF (**c**). Black lines represent the tablets as produced, grey after 5 cycles, brown after 15 (PA11) or 20 cycles. Box plots represent the maximum compression force measured for four independent tablets for each sample.

The mechanical properties of these polymer-containing composites were evaluated using compression tests (Figure 6).

All formulations exhibited enhanced mechanical stability compared to their salt-only counterparts. However, the differences in performance among the polymers became more pronounced during cycling. For instance, K_2_CO_3_/PA11 tablets (Figure 6a) demonstrated notable resilience immediately after production but showed significant strength degradation after five and ten cycles. This deterioration can be linked to the limited alkaline resistance of PA11, which may undergo hydrolytic degradation, compromising the network integrity [32].

In contrast, K_2_CO_3_/PEI tablets (Figure 6b) maintained their mechanical properties relatively well, with less pronounced reductions observed after five cycles. However, by twenty cycles, a more pronounced decrease in strength was noted.

K_2_CO_3_/PVDF tablets (Figure 6c), on the other hand, exhibited deformation and relaxation behavior, remaining intact after force removal even after twenty cycles, indicating a more robust performance under cyclic conditions. A similar behavior was reported for PDMS-based flexible matrices [16]; however, the strength level of approximately 200 N initially and about 80 N after 20 cycles represents a noticeable improvement in this case.

**Figure 7:**
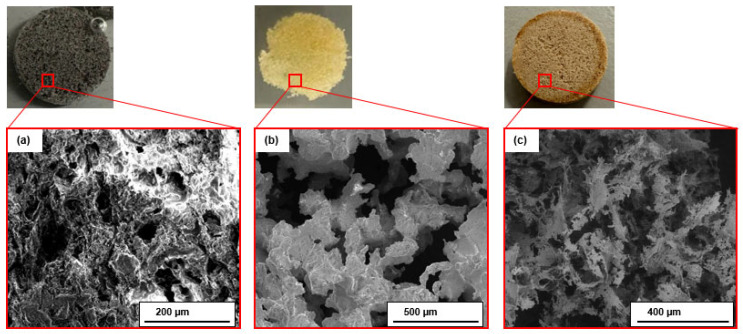
SEM of polymeric networks (**a**) PA11 after 5 cycles, (**b**) PEI after 20 cycles, (**c**) PVDF after 20 cycles.

If we look at the SEM pictures of the the polymeric networks when they were just formed in the tablets, as described in Section 2.2, followed by dissolving the salt, we can see differences between the networks formed by different polymers (Figure 7). Dissolving salt from polymer/salt tablets allows to reveal polymeric network the way it formed within tablet structure. The structure of PVDF network contains smaller/thinner parts compared to the other two polymers as can be seen from the SEM images (Figure 7).

**Figure 8:**
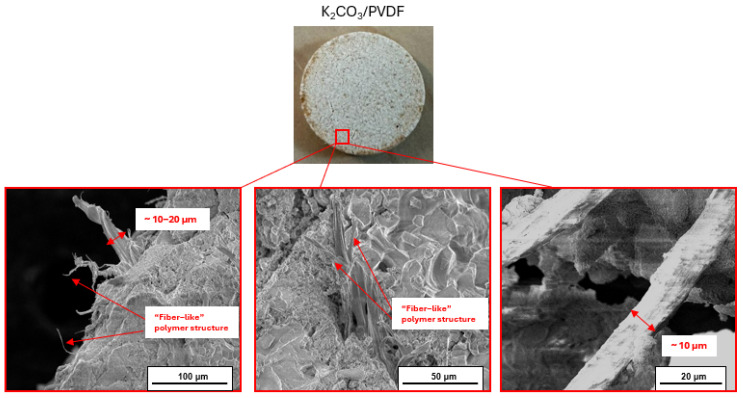
SEM images of PVDF network.

The distinctive structural features of the PVDF network, characterized by smaller and thinner filaments compared to PA11 and PEI, likely contributed to its mechanical performance (Figure 8).

These “fiber-like” structures are formed after the polymer is melted during the preparation process, at which point the polymeric network takes shape. Upon cooling, the resulting filaments enhance the incorporation of the polymer into the salt matrix, providing a higher stability [17,36]. This can explain the behavior of PVDF composite during compression test after cycling (Figure 7c). The polymeric network can act as the primary load-bearing element. When a composite is subjected to mechanical stress, the load is efficiently transferred from the weaker matrix to the soft elastic polymer structure. This load transfer enhances the overall strength.

The SEM images presented in this study were analyzed qualitatively to demonstrate the morphology of the polymeric network, the distribution of the polymer phase. Quantitative morphological parameters, such as pore size distribution, polymer domain dimensions, or interconnectivity, were not determined, as the main objective of this work was to establish a proof of concept for network formation and its effect on mechanical stability over hydration/dehydration cycling. Nonetheless, such quantitative image analysis could be valuable for future investigations to provide a more detailed correlation between the structural characteristics of the polymer network and the resulting mechanical and kinetic behavior.

Polymers with higher elongation at break, such as PVDF and PA11, form a compliant network that accommodates swelling of the salt particles, absorbing strain energy and preventing fracture. However, PA11 gradually disintegrates over cycling due to higher water uptake. The fiber-like structures of PVDF (Figure 7 and Figure 8) act as flexible bridges within the salt tablet, distributing stress during volumetric expansion and reducing crack propagation. In contrast, more rigid polymers like PEI create localized reinforcement that limits expansion while preserving overall tablet integrity. SEM observations reveal that PVDF fibers and PA11 domains form continuous, interconnected networks, whereas PEI forms discrete networks providing localized support. These morphological features correlate with mechanical stability and cyclic performance observed in compression tests and hydration kinetics, demonstrating that polymer network architecture and mechanical properties jointly govern tablet durability under repeated hydration–dehydration cycles.

The stability of tablets over cycling was investigated by placing dry single tablets of both K_2_CO_3_ and K_2_CO_3_/polymer in a desiccator over a saturated MgCl_2_ solution (relative humidity 33% at 20 °C [25]) to induce salt hydration. The hydration rate was monitored during this phase of the cycle. For dehydration kinetics measurements, fully hydrated tablets were transferred to an oven at 130 °C instead of the desiccator.

**Figure 9:**
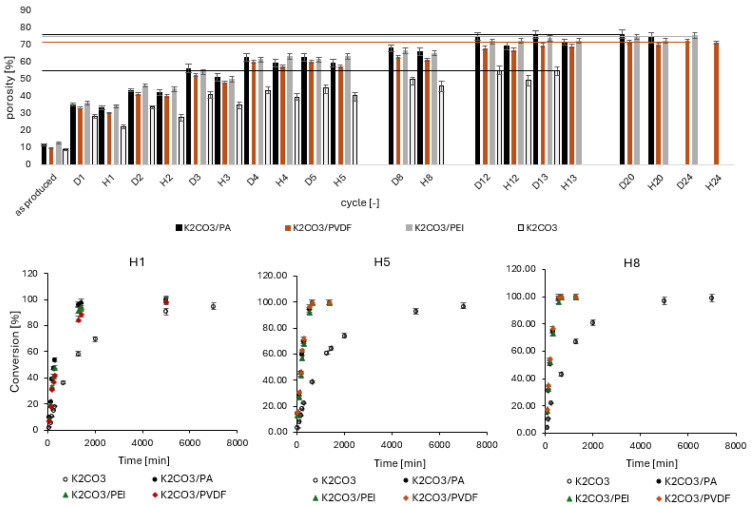
Porosity level of tablets K_2_CO_3_, K_2_CO_3_/PA11_10 wt%, K_2_CO_3_/PEI_10 wt%, K_2_CO_3_/PVDF_10 wt% over cycling, where “D” means dehydration and “H” means hydration followed by the corresponding number of cycle (top row); Hydration rate of K_2_CO_3_, K_2_CO_3_/PA11_10 wt%, K_2_CO_3_/PEI_10 wt%, K_2_CO_3_/PVDF_10 wt% tablets over cycling (bottom row). Each data point represents the mean of four tablets.

Throughout cycling, changes in tablet mass and dimensions were tracked to assess porosity evolution and structural integrity. Figure 9 presents the porosity changes in pure salt and salt/polymer tablets (prepared from powder fraction ≤ 300 µm) over multiple cycles, alongside the corresponding hydration rates for some of the cycles. The lines in Figure 9 indicate the maximum porosity level at which the tablets remain intact. Notably, while polymer-incorporated tablets can sustain significantly higher porosity levels while remaining structurally stable, pure salt tablets do not reach such high porosity before disintegrating.

A marked increase in porosity is evident after the second and third cycle across all three salt/polymer systems. This increase in porosity can likely be attributed to the swelling and shrinking behavior of the salt embedded within the polymer matrix. Unlike the salt, the polymer itself does not undergo significant swelling or shrinking, leading to the formation of additional voids early in the cycling process. The mismatch in the expansion and contraction behaviors of the salt and polymer contributes to the development of these voids.

The observed increase in porosity is consistent with a corresponding rise in the hydration rate for all salt/polymer systems tested, suggesting that the structural changes within the matrix facilitate greater water uptake. A similar trend was observed in studies employing PDMS matrices [16]; however, unlike those systems, the volume increase in the present work slows down significantly and stabilizes after approximately 10–12 cycles.

It was observed that the hydration rate increased significantly after the first cycle for all polymer-incorporated tablets, with porosity levels rising from an initial 10% to around 37% after the first cycle, and to 65% after five cycles. In contrast, pure K_2_CO_3_ tablets did not exhibit such hydration rate increase. This behavior contrasts with previous encapsulation-based studies, where an increased polymer content typically led to slower hydration kinetics [19].

**Figure 10:**
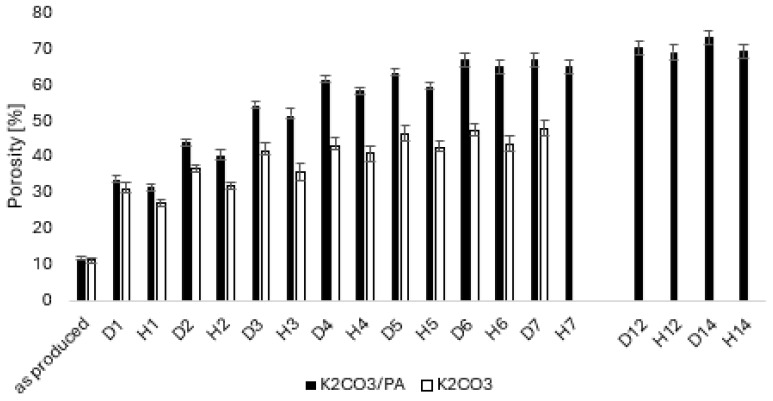
Porosity level of tablets K_2_CO_3_, K_2_CO_3_/PA11_10 wt% prepared using 300–500 µm fraction of K_2_CO_3_.

A set of tablets prepared from the 300–500 µm powder fraction, including pure K_2_CO_3_ tablets and K_2_CO_3_/PA11 (10 wt%) composites, was tested for stability over multiple cycles (Figure 10).

The results indicated that tablets incorporating polymer exhibited greater durability and maintained a higher porosity level throughout the cycling process. However, pure salt tablets prepared from the 300–500 µm fraction remained intact for fewer cycles compared to those made from the ≤300 µm fraction. This can be attributed to the increased contact points and enhanced particle packing in the finer fraction (≤300 µm), which likely improves mechanical stability and cohesion within the tablet. In contrast, the coarser fraction (300–500 µm) may result in weaker interparticle bonding, making the tablets more prone to fragmentation and degradation over repeated cycles. This aspect would be interesting for a separate further investigation into the effects of particle packing and porosity, which could provide valuable insights for optimizing material performance.

Despite this high level of porosity (above 70%), the potassium carbonate/polymer tablets remained structurally intact throughout the cycling tests. These findings highlight the stabilizing effect of incorporating a polymer matrix, which enhances the mechanical resilience of the tablets and allows them to withstand the stresses associated with repeated cycling, even at elevated porosity levels. This demonstrates the potential of salt/polymer composites for applications requiring high porosity and stability under cyclic conditions. It is important to note that the specific polymers used in this study were selected as model systems to investigate which polymer properties could be beneficial for this application, rather than to identify a single optimal polymer for practical use. This demonstrates the potential of salt/polymer composites for applications, while also suggesting that further polymer selection and optimization could enhance performance even further.

Although a detailed economic analysis was not conducted in this study, the proposed solvent-free melt-processing method developed in this study presents a promising pathway for large-scale fabrication of mechanically stable salt–polymer composites. The use of standard thermoplastic polymers and compression or extrusion techniques allows for scalability without complex chemical processing. Considering bulk material costs, K_2_CO_3_ is typically available at ~2–2.5 EUR/kg, while thermoplastic polymers such as PA11, PEI, and PVDF range roughly from 6 to 35 EUR/kg depending on grade and source. With polymer content limited to 10 wt%, the material cost contribution remains moderate. Future work may focus on optimizing polymer selection, content, and processing conditions to enhance economic performance. Such techniques as compression molding, tableting, or extrusion are already well established in polymer and pharmaceutical manufacturing, suggesting that the polymer-stabilized salt hydrate composites could be produced at large scale with relatively low adaptation costs. Additionally, the melt-based fabrication route avoids the need for solvents or complex chemical reactions, reducing process complexity and environmental impact. Cost considerations suggest that low-cost, recyclable thermoplastics or blends thereof could offer favorable techno-economic performance compared with microencapsulation or porous matrix methods. Integrating this approach into continuous manufacturing systems could accelerate industrial deployment of polymer-stabilized thermochemical storage materials.

## 4. Conclusions

This work explored an alternative approach to stabilize salt tablets using thermoplastic polymer networks. Polyamide 11 (PA-11), polyetherimide (PEI) and polyvinylidene fluoride (PVDF) were investigated for their suitability. The addition of PA-11 and PVDF led to the formation of a flexible network within the tablet structure, while PEI exhibited rigidity. All polymers demonstrated K_2_CO_3_ tablets improved stability at relatively high level of porosity (above 70%). The hydration kinetics of tablets with integrated polymer network is up to 5 times faster than pure K_2_CO_3_.

The PVDF network showed superior performance, particularly due to its elastic behavior and resilience after at least 24 cycles. This highlights not the specific polymer performance, but the potential of polymers with similar properties for enhancing the mechanical stability and long-term performance of salt hydrates. The polymeric network approach, formed by melting polymer within the salt tablet, proves to be a potentially scalable method due to its simplicity in tablet formation, making it promising for industrial applications.

The cycling experiments in this study were performed over at least 24 hydration/dehydration cycles in the case of the PVDF network, which was sufficient to identify degradation trends and differences among the polymer networks. While PVDF in particular demonstrated stable performance within this range, extended testing over 50–100 cycles will be necessary in future studies to fully assess long-term mechanical integrity and possible chemical aging under repeated cycling conditions relevant to industrial TCES applications.

While the present study was conducted under controlled laboratory conditions to provide a consistent basis for comparison, future investigations may focus on testing the materials under realistic TCES operating environments, including variable humidity, temperature cycling, and vapor flow rates, to evaluate the applicability and long-term stability of the polymer-stabilized salt composites in practical systems.

The approach of forming a polymeric network by melting polymer withing salt tablet can be a potentially scalable method due to simplicity of tablet formations, which makes it promising for industrial application.

While this study highlights the promise of polymer-based stabilization, further research into optimizing polymer selection and understanding the interactions between salt hydrates and polymers could further improve the performance and scalability of TCES systems. Additionally, the choice of polymer may vary depending on specific application needs, such as chemical resistance, flexibility, and environmental considerations.

## Figures and Tables

**Table 1:** Selected polymers’ characteristics.

	Melting Point	Elongation at Break	Water Uptake
Polyamide (nylon) 11 (PA-11)	200 °C	300%	1–2%
Polyetherimide (PEI)	230 °C * (315–320 °C) **	50%	≤0.5%
Polyvinylidene fluoride (PVDF)	180 °C * (250 °C) **	10–100%	0.1–2%

* The melting temperature for polymers according to literature [31,32,33,34,35]. ** The temperature selected empirically based on the state where the polymer powder reaches liquid state providing homogeneous liquid able to form network in the tablet pores.

## Data Availability

The original contributions presented in this study are included in the article. Further inquiries can be directed to the corresponding author.

## References

[1] Mohapatra D., Nandanavanam J. (2023). Salt in matrix for thermochemical energy storage—A review. Mater. Today Proc..

[2] Kiyabu S., Girard P., Siegel D.J. (2022). Discovery of salt hydrates for thermal energy storage. J. Am. Chem. Soc..

[3] Donkers P.A.J., Sögütoglu L.C., Huinink H.P., Fischer H.R., Adan O.C.G. (2016). A review of salt hydrates for seasonal heat storage in domestic applications. Appl. Energy.

[4] Kenisarin M., Mahkamov K. (2016). Salt hydrates as latent heat storage materials: Thermophysical properties and costs. Sol. Energy Mater. Sol. Cells.

[5] Cabeza L.F., Solé C., Castell A. (2011). Thermal energy storage using phase change materials in solar cooling and refrigeration. Sol. Energy.

[6] N’Tsoukpoe K.E., Liu H., Le Pierrès N., Luo L. (2009). A review on long-term sorption solar energy storage. Renew. Sustain. Energy Rev..

[7] Pardo P., Deydier A., Anxionnaz-Minvielle Z., Rougé S., Cabassud M., Cognet P. (2014). A review on high temperature thermochemical heat energy storage. Renew. Sustain. Energy Rev..

[8] Sögütoglu L.C., Donkers P.A.J., Fischer H.R., Huinink H.P., Adan O.C.G. (2018). In-depth investigation of thermochemical performance in a heat battery: Cyclic analysis of K_2_CO_3_, MgCl_2_ and Na_2_S. Appl. Energy.

[9] Aarts J., Fischer H., Adan O., Huinink H. (2024). Impact of cycling on the performance of mm-sized salt hydrate particles. J. Energy Storage.

[10] Shkatulov A.I., Houben J., Fischer H., Huinink H.P. (2020). Stabilization of K_2_CO_3_ in vermiculite for thermochemical energy storage. Renew. Energy.

[11] Aydin D., Casey S.P., Riffat S. (2015). The latest advancements on thermochemical heat storage systems. Renew. Sustain. Energy Rev..

[12] Jabbari-Hichri A., Bennici S., Auroux A. (2017). CaCl_2_-containing composites as thermochemical heat storage materials. Sol. Energy Mater. Sol. Cells.

[13] Brancato V., Calabrese L., Palomba V., Frazzica A., Fullana-Puig M., Solé A., Cabeza L.F. (2018). MgSO_4_·7H_2_O filled macro cellular foams: An innovative composite sorbent for thermo-chemical energy storage applications for solar buildings. Sol. Energy.

[14] Aarts J., van Ravensteijn B., Fischer H., Adan O., Huinink H. (2023). Polymeric stabilization of salt hydrates for thermochemical energy storage. Appl. Energy.

[15] Shkatulov A., Joosten R., Fischer H., Huinink H. (2020). Core–Shell Encapsulation of Salt Hydrates into Mesoporous Silica Shells for Thermochemical Energy Storage. ACS Appl. Energy Mater..

[16] Aarts J., van Ravensteijn B., Fischer H., Adan O., Huinink H. (2023). Stabilization of salt hydrates using flexible polymeric networks. Energy.

[17] Shkatulov A., Averina E., Raemaekers T., Fischer H., Adan O.C.G., Huinink H. (2024). Stabilization of reactive bed particles for thermochemical energy storage with fiber reinforcement. J. Energy Storage.

[18] Graham M., Shchukina E., De Castro P.F., Shchukin D. (2016). Nanocapsules containing salt hydrate phase change materials for thermal energy storage. J. Mater. Chem. A.

[19] Van Ravensteijn B.G., Donkers P.A., Ruliaman R.C., Eversdijk J., Fischer H.R., Huinink H.P., Adan O.C. (2021). Encapsulation of salt hydrates by polymer coatings for low-temperature heat storage applications. ACS Appl. Polym. Mater..

[20] Elahi B., Salehzadeh D., de Vos W.M., Shahidzadeh N., Brem G., Mehrali M. (2024). Boosting stability of K_2_CO_3_ granules for thermochemical heat storage applications through innovative membrane encapsulation. Chem. Eng. J..

[21] Oliver-Ortega H., Méndez J.A., Mutjé P., Tarrés Q., Espinach F.X., Ardanuy M. (2017). Evaluation of thermal and thermomechanical behaviour of bio-based polyamide 11 based composites reinforced with lignocellulosic fibres. Polymers.

[22] Lods L., Richmond T., Dandurand J., Dantras E., Lacabanne C., Durand J.M., Ponteins P. (2022). Continuous bamboo fibers/fire-retardant polyamide 11: Dynamic mechanical behavior of the biobased composite. Polymers.

[23] Wypych G. (2021). Handbook of Polymers for Electronics.

[24] Serfaty I.W. (2020). Polyetherimide. Engineering Thermoplastics.

[25] Greenspan L. (1977). Humidity fixed points of binary saturated aqueous solutions. J. Res. Natl. Bur. Stand. Sect. A Phys. Chem..

[26] Van Helden W. (2023). Compact Thermal Energy Storage.

[27] Heating—Fuels & Technologies. IEA n.d. https://www.iea.org/fuels-and-technologies/heating.

[28] Fu S.Y., Feng X.Q., Lauke B., Mai Y.W. (2008). Effects of particle size, particle/matrix interface adhesion and particle loading on mechanical properties of particulate–polymer composites. Compos. Part B Eng..

[29] Jariyavidyanont K., Focke W., Androsch R. (2019). Thermal properties of biobased polyamide 11. Thermal Properties of Bio-Based Polymers.

[30] Tey W.S., Cai C., Zhou K. (2021). A comprehensive investigation on 3D printing of polyamide 11 and thermoplastic polyurethane via multi jet fusion. Polymers.

[31] Malotky D.L., Dermody D.L., Schmidt D., Young T.J., Kalinowski M. (2019). Development, Characterization, and Application of Novel High Temperature Thermoplastic and Thermosetting Dispersions. Polymer Colloids: Formation, Characterization and Applications.

[32] Puhan M.R., Sutariya B., Karan S. (2022). Revisiting the alkali hydrolysis of polyamide nanofiltration membranes. J. Membr. Sci..

[33] Huang J., Luo J., Chen X., Feng S., Wan Y. (2021). New insights into effect of alkaline cleaning on fouling behavior of polyamide nanofiltration membrane for wastewater treatment. Sci. Total Environ..

[34] Relles H.M. (1984). Synthesis and Properties of Polyetherimide Polymers. Contemporary Topics in Polymer Science: Volume 5.

[35] Wypych G. (2022). Handbook of Polymers.

[36] Karger-Kocsis J., Mahmood H., Pegoretti A. (2015). Recent advances in fiber/matrix interphase engineering for polymer composites. Prog. Mater. Sci..

